# Placebos without Deception: A Randomized Controlled Trial in Irritable Bowel Syndrome

**DOI:** 10.1371/journal.pone.0015591

**Published:** 2010-12-22

**Authors:** Ted J. Kaptchuk, Elizabeth Friedlander, John M. Kelley, M. Norma Sanchez, Efi Kokkotou, Joyce P. Singer, Magda Kowalczykowski, Franklin G. Miller, Irving Kirsch, Anthony J. Lembo

**Affiliations:** 1 Beth Israel Deaconess Medical Center, Harvard Medical School, Boston, Massachusetts, United States of America; 2 Osher Research Center, Harvard Medical School, Boston, Massachusetts, United States of America; 3 Psychology Department, Endicott College, Beverly, Massachusetts, United States of America; 4 Massachusetts General Hospital, Harvard Medical School, Boston, Massachusetts, United States of America; 5 Department of Bioethics, National Institutes of Health, Bethesda, Maryland, United States of America; 6 Department of Psychology, University of Hull, Hull, United Kingdom; University Paris Descartes, France

## Abstract

**Background:**

Placebo treatment can significantly influence subjective symptoms. However, it is widely believed that response to placebo requires concealment or deception. We tested whether open-label placebo (non-deceptive and non-concealed administration) is superior to a no-treatment control with matched patient-provider interactions in the treatment of irritable bowel syndrome (IBS).

**Methods:**

Two-group, randomized, controlled three week trial (August 2009-April 2010) conducted at a single academic center, involving 80 primarily female (70%) patients, mean age 47±18 with IBS diagnosed by Rome III criteria and with a score ≥150 on the IBS Symptom Severity Scale (IBS-SSS). Patients were randomized to either open-label placebo pills presented as “placebo pills made of an inert substance, like sugar pills, that have been shown in clinical studies to produce significant improvement in IBS symptoms through mind-body self-healing processes” or no-treatment controls with the same quality of interaction with providers. The primary outcome was IBS Global Improvement Scale (IBS-GIS). Secondary measures were IBS Symptom Severity Scale (IBS-SSS), IBS Adequate Relief (IBS-AR) and IBS Quality of Life (IBS-QoL).

**Findings:**

Open-label placebo produced significantly higher mean (±SD) global improvement scores (IBS-GIS) at both 11-day midpoint (5.2±1.0 vs. 4.0±1.1, *p*<.001) and at 21-day endpoint (5.0±1.5 vs. 3.9±1.3, *p* = .002). Significant results were also observed at both time points for reduced symptom severity (IBS-SSS, *p* = .008 and *p* = .03) and adequate relief (IBS-AR, *p* = .02 and *p* = .03); and a trend favoring open-label placebo was observed for quality of life (IBS-QoL) at the 21-day endpoint (*p* = .08).

**Conclusion:**

Placebos administered without deception may be an effective treatment for IBS. Further research is warranted in IBS, and perhaps other conditions, to elucidate whether physicians can benefit patients using placebos consistent with informed consent.

**Trial Registration:**

ClinicalTrials.gov NCT01010191

## Introduction

Placebo treatment can have a significant impact on subjective complaints. [1] Furthermore, recent studies have shown measurable physiological changes in response to placebo treatment that could explain how placebos alter symptoms. [2] A critical question is establishing how physicians and other providers can take optimal advantage of placebo effects consistent with their responsibility to foster patient trust and obtain informed consent. Directly harnessing placebo effects in a clinical setting has been problematic because of a widespread belief that beneficial responses to placebo treatment require concealment or deception. [3] This belief creates an ethical conundrum: to be beneficial in clinical practice placebos require deception but this violates the ethical principles of respect for patient autonomy and informed consent. In the clinical setting, prevalent ethical norms emphasize that “the use of a placebo without the patient's knowledge may undermine trust, compromise the patient-physician relationship, and result in medical harm to the patient.” [4] Nevertheless, a recent national survey of internists and rheumatologists in the US found that while only small numbers of US physicians surreptitiously use inert placebo pills and injections, approximately 50% prescribe medications that they consider to have no specific effect on patients' conditions and are used solely as placebos (sometimes called “impure placebos.”) [5] Many other studies confirm this finding. [6] Given this situation, finding effective means of harnessing placebo responses in clinical practice without deception is a high priority.

Irritable bowel syndrome (IBS) is one of the top 10 reasons for seeking primary care and with a world-wide prevalence of approximately 10 to 15%. [7], [8] It is a chronic functional gastrointestinal disorder characterized by abdominal pain and discomfort associated with altered bowel habits. [9] The symptoms of IBS not only adversely affect a person's health-related quality of life (QOL), [10], [11] but are associated with a substantial financial burden of reduced work productivity and an over 50% increase in the use of health-related resources. [11], [12] While many therapies are commonly used to treat individual IBS symptoms such as constipation or diarrhea, few therapies have been shown to be effective and safe in relieving the global symptoms of IBS. [11], [13] Previous research has demonstrated that placebo responses in IBS are substantial and clinically significant. [14], [15] Furthermore, data from our previous qualitative study of IBS patients being treated single-blind with placebos indicated that patients can tolerate a high degree of ambiguity and uncertainty about placebo treatment and still benefit. [16] In view of these considerations, we selected IBS as a suitable condition to test the widespread belief that placebo responses are neutralized by awareness or knowledge that the treatment is a placebo.

The objectives of this study were to assess the feasibility of recruiting IBS patients to participate in a trial of open-label placebo and to assess whether an open-label placebo pill with a persuasive rationale was more effective than no-treatment in relieving symptoms of IBS in the setting of matched patient-provider interactions.

## Methods

### Design

A three week randomized controlled trial (RCT) comparing open-label placebo to no-treatment controls was conducted between August 2009 and April 2010 in a single academic medical center. Written informed consent was obtained from each patient prior to participation on the study. The Beth Israel Deaconess Medical Center Institutional Review Board approved the design and informed consent.

Patients who gave informed consent and fulfilled the inclusion and exclusion criteria were randomized into two groups: 1) placebo pill twice daily or 2) no-treatment. Before randomization and during the screening, the placebo pills were truthfully described as inert or inactive pills, like sugar pills, without any medication in it. Additionally, patients were told that “placebo pills, something like sugar pills, have been shown in rigorous clinical testing to produce significant mind-body self-healing processes.” The patient-provider relationship and contact time was similar in both groups. Study visits occurred at baseline (Day 1), midpoint (Day 11) and completion (Day 21). Assessment questionnaires were completed by patients with the assistance of a blinded assessor at study visits. (The protocol for this trial and supporting CONSORT checklist are available as supporting information; see Checklist S1 and Protocol S1.)

### Patients

Participants were recruited from advertisements for “a novel mind-body management study of IBS” in newspapers and fliers and from referrals from healthcare professionals. During the telephone screening, potential enrollees were told that participants would receive “either placebo (inert) pills, which were like sugar pills which had been shown to have self-healing properties” or no-treatment. Participants were adults (≥18 years old) meeting the Rome III criteria for IBS [17] with a score of ≥150 on the IBS Symptom Severity Scale (IBS-SSS). [18] The diagnosis of IBS was based on typical symptoms and exclusion of patients with alarm symptoms. [19], [20] was confirmed by a board certified gastroenterologist (AJL) or a nurse practitioner (EF) experienced in functional bowel disorders. Patients were excluded if they had any unexplained alarm features (i.e. weight loss >10% body weight, fevers, or blood in stools, or had family history of colon cancer, or inflammatory bowel disease). Patients with a history of pelvic floor dyssynergia, the need to use manual maneuvers in order to achieve a bowel movement, surgery of the colon at any time, abdominal surgery within 60 days prior to entry into the study, or laxative abuse were excluded from the study. Patients with other medical conditions (e.g., neurological disorders, metabolic disorders, or other significant disease), or pretreatment laboratory or ECG findings believed to impair their ability to participate in the study were also excluded. Any surgery within the past 30 days, pregnancy, breast-feeding, or participation in another clinical study within 30 days prior to the start of the study were also disqualifying factors.

Patients were allowed to continue IBS medications (e.g., fiber, anti-spasmodics, loperamide, etc.) as long as they had been on stable doses for at least 30 days prior to entering the study and agreed not to change medications or dosages during the trial. Patients were asked to refrain from making any major life-style changes (e.g., starting a new diet or changing their exercise pattern) during the study.

### Interventions

Patients were randomly assigned either to open-label placebo treatment or to the no-treatment control. Prior to randomization, patients from both groups met either a physician (AJL) or nurse-practitioner (EF) and were asked whether they had heard of the “placebo effect.” Assignment was determined by practitioner availability. The provider clearly explained that the placebo pill was an inactive (i.e., “inert”) substance like a sugar pill that contained no medication and then explained in an approximately fifteen minute *a priori* script the following “four discussion points:” 1) the placebo effect is powerful, 2) the body can automatically respond to taking placebo pills like Pavlov's dogs who salivated when they heard a bell, 3) a positive attitude helps but is not necessary, and 4) taking the pills faithfully is critical. Patients were told that half would be assigned to an open-label placebo group and the other half to a no-treatment control group. Our rationale had a positive framing with the aim of optimizing placebo response. It was emphasized that each group was critical for the trial. All patients were told that they would receive educational recommendations for their IBS at the end of the study. After completion of the physical examination and assessments, patients were then randomized using a sequentially numbered opaque sealed envelopes that contained treatment assignments drawn from a computer-generated random number sequence. Until this point, the patient-provider interaction --- including delivering the persuasive rationale and the explanation of the importance of both groups – was similar for all participants. At this point, during the last moments of the interview, they were told their assignments. Patients randomized to the open-label placebo group were given a typical prescription medicine bottle of placebo pills with a label clearly marked “placebo pills” “take 2 pills twice daily.” The placebo pills were blue and maroon gelatin capsules filled with avicel, a common inert excipient for pharmaceuticals (Bird's Hill Pharmacy, Needham, MA). Patients in the no treatment arm were reminded of the importance of the control arm. All visits were in the context of a warm supportive patient-practitioner relationship. The midpoint 11 day visit was brief (approximately 15 minutes) and included an opened question regarding adverse events, concomitant medications and a brief physical examination. After the examination, a treatment-blind researcher administered questionnaires. Patients receiving placebos received a short reminder regarding the “four discussion points.” In the no treatment arm, patients were encouraged and thanked for helping make a successful study.

Before the study began the providers practiced the trial procedures on simulated and real patients. Once a month, the two providers (AJL, EF) and a third researcher (TJK) met to discuss fidelity to the protocol and any other problems. AJL and EF consistently reported that they had no problem holding the entire initial interview process to approximately 30 minutes and the mid-point to 15 minutes.

### Assessment

Our primary outcome measure was the IBS Global Improvement Scale (IBS-GIS) which asks participants: “Compared to the way you felt before you entered the study, have your IBS symptoms over the past 7 days been: 1) Substantially Worse, 2) Moderately Worse, 3) Slightly Worse, 4) No Change, 5) Slightly Improved, 6) Moderately Improved or 7) Substantially Improved.[21], [22] Other measures included: the IBS-SSS measure, which contains five 100-point scales, that assess the severity of abdominal pain, the frequency of abdominal pain, the severity of abdominal distention, dissatisfaction with bowel habits, and interference with quality of life, [18] All 5 components contribute to the score equally yielding a theoretical range of 0–500, with a higher score indicating greater symptom severity. The IBS-Adequate Relief (IBS-AR) is a single dichotomous (yes or no) item that asks participants “Over the past week have you had adequate relief of your IBS symptoms?” [23] The IBS-QoL is a 34-item measure assessing the degree to which IBS interferes with patient quality of life. Each item is rated on a 5-point Likert scale and a linear transformation yields a summed score with a theoretical range of 0 to 100, with a higher score indicating better quality of life. [24] Side effects were recorded at each assessment. A pill count was taken at visits two and three. Given the unprecedented nature of the study, at the completion of the trial patients were given a short qualitative open-ended check-out questionnaire and asked for written responses. The questions were different for each group. Those in the placebo treatment arm were asked four questions: What do you think of about the idea of taking placebo? Did you expect it to work or were you skeptical? What did you think was in the placebo pills? Any further comments? Those in the no-treatment were asked three questions: Were you disappointed to be in the treatment as usual arm? What did you like most and least about the trial? Any further comments? All assessments were performed by a researcher who was blind to treatment assignment.

### Statistical Analysis

All tests were two-tailed with alpha set at .05. All results are reported as mean ±SD unless otherwise noted. All analyses were intent-to-treat, and missing data were replaced using the last observation carried forward method. Since IBS-GIS and IBS-AR are change scores and are not assessed at baseline, we carried forward scores for patients who had at least one follow-up visit. For our main outcome measure (IBS-GIS at 21-day endpoint), we planned an independent samples t-test. We estimated *a priori* that a total sample size of 80 would provide 94% power to detect a large effect (d = .8) and 60% power to detect a medium effect (d = .5). For IBS-SSS and IBS-QOL, we computed change scores from baseline and then conducted independent samples t-tests. We used chi square tests of independence for IBS-AR. Per protocol analyses were also conducted, but they produced no substantive differences from our planned intent-to-treat analyses and are not reported here.

## Results

**Figure 1:**
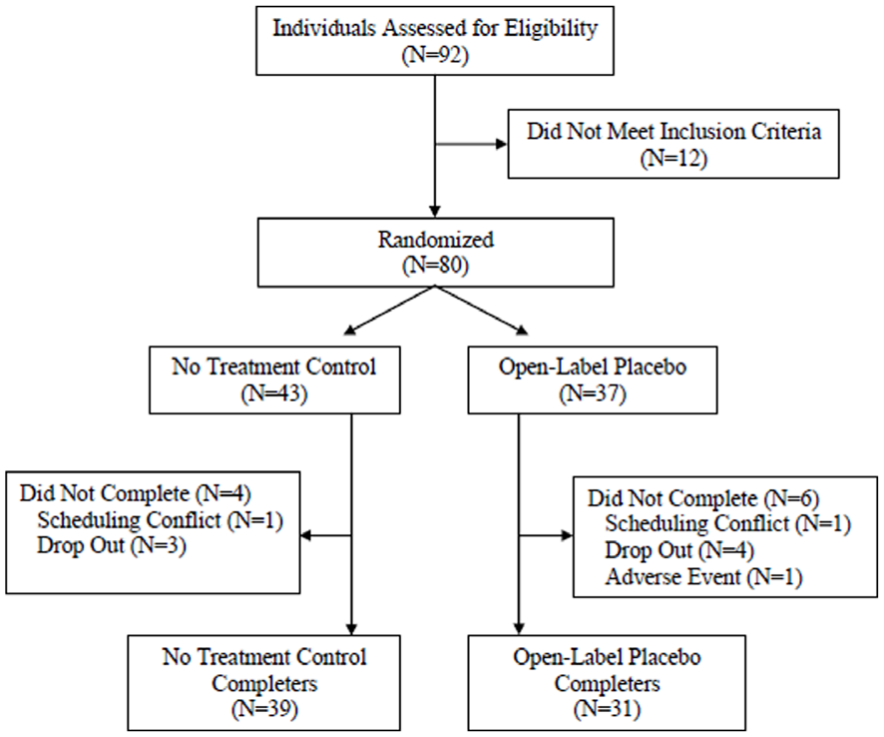
Enrollment Flowchart.

As shown in Figure 1, 92 patients were screened, and 80 eligible patients were randomized into the two arms (43 into no-treatment and 37 into open-label placebo). There were missing outcome data for 13 patients at midpoint (16%; 6 no-treatment control, 7 open-label placebo), and for 10 patients at endpoint (13%; 4 no-treatment control, 6 open-label placebo). As noted above, missing data was replaced using the last observation carried forward method. Table 1 shows baseline data.

**Table 1:** Demographics and Baseline Characteristics.

Demographics and Baseline Characteristics	No Treatment(N = 43)	Open Placebo(N = 37)
Age	46±18	47±18
Female – no. (%)	32 (74)	24 (65)
White – no. (%)	36 (84)	26 (70)
IBS Type – no. (%)		
Diarrhea Predominant	16 (37)	10 (27)
Constipation Predominant	14 (33)	16 (43)
Mixed	13 (30)	11 (30)
IBS Duration in Years	13±11	16±12
Symptom Severity (IBS-SSS)	297±58	310±82
Quality of Life (IBS-QOL)	59±21	55±21
Upper GI Symptoms (GERD & Dyspepsia) – no. (%)	18 (42)	11 (30)
Taking Medications for IBS – no. (%)	15 (35)	20 (54)
Taking Antidepressants – no. (%)	7 (16)	9 (24)

**Note**: All values are means ±SD, unless otherwise noted. Group differences were examined using independent t-tests for continuous measures and chi square test for categorical measures.. IBS  =  irritable bowel syndrome; IBS-SSS  =  IBS Symptom Severity Scale; IBS-QOL  =  IBS Quality of Life Scale; GI  =  Gastrointestinal; GERD  =  Gastroesophageal Reflux Disease.

**Figure 2:**
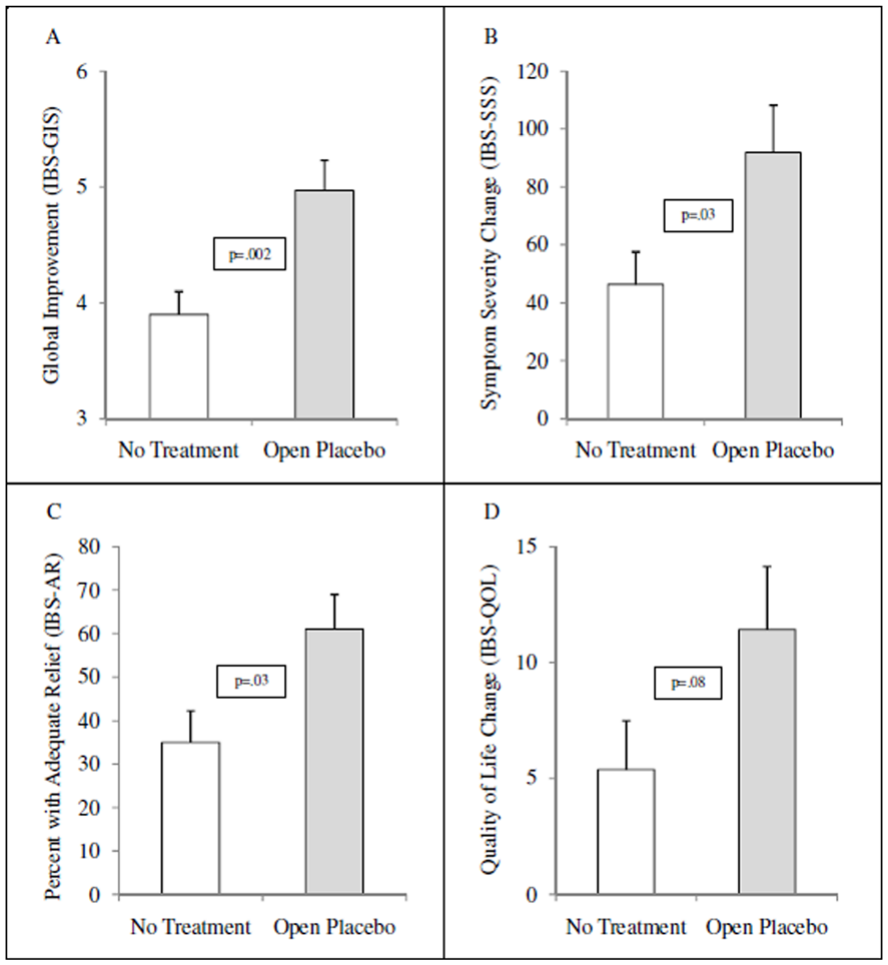
Outcomes at the 21-Day Endpoint by Treatment Group.

As shown in Figure 2 and Table 2, patients treated with open-label placebo had significantly greater scores than the no-treatment control on the main outcome measure, Global Improvement Scale (IBS-GIS), at both the 11-day midpoint (5.2±1.0 vs. 4.0±1.1, *p*<.001, *d* = 1.14) and the 21-day endpoint (5.0±1.5 vs. 3.9±1.3, *p* = .002, *d* = 0.79). In addition, there were statistically significant differences at both time points on reduction on in symptom severity (IBS-SSS) and adequate relief (IBS-AR), and a trend toward significance at the 21-day endpoint on improvement in quality of life (IBS-QOL).

**Table 2:** Treatment Outcomes.

	No Treatment(N = 43)	Open Placebo(N = 37)	p-value
**Midpoint (11 Days)**			
Global Improvement (IBS-GIS)	4.0±1.1	5.2±1.0	<.001
Adequate Relief (IBS-AR) – no. (%)	10 (23)	18 (49)	.02
Symptom Severity Reduction (IBS-SSS)	28±66	75±87	.008
Quality of Life Improvement (IBS-QoL)	4.4±8.9	8.3±11.6	.10
**Endpoint (3 Weeks)**			
Global Improvement (IBS-GIS)	3.9±1.3	5.0±1.5	.002
Adequate Relief (IBS-AR) – no. (%)	15 (35)	22 (59)	.03
Symptom Severity Reduction (IBS-SSS)	46±74	92±99	.03
Quality of Life Improvement (IBS-QoL)	5.4±13.8	11.4±16.6	.08

**Note**: All values are means ±SD except where noted. IBS  =  irritable bowel syndrome; IBS-GIS  =  IBS Global Improvement Scale; IBS-AR  =  IBS Adequate Relief; IBS-SSS  =  IBS Symptom Severity Scale; IBS-QoL  =  IBS Quality of Life Scale.

Forty-three patients saw the male physician for all three visits, 20 patients saw the female nurse-practitioner for all three visits, and 17 patients saw a combination of the two or missed a treatment session. Given that the two treatment providers differed by gender and discipline (MD vs. NP), we tested for differences in treatment outcomes. No significant differences were found between providers on the primary outcome measure, IBS-GIS (p = .57 at midpoint, and p = .51 at endpoint). Similarly, there were no significant differences between providers on any of the secondary outcome measures.

Adverse events were reported by only three placebo-treated patients (8%) at midpoint and five patients (14%) at endpoint. The most common adverse events that patients reported were upper respiratory infection (N = 3) and pain (N = 2); other events included rash, runny stools, and a sty on the eye.

The detailed results of the qualitative check-out questionnaire will be reported elsewhere. However, responses to two questions seemed especially relevant to the interpretation of this quantitative report. Specifically, 1) did patients in the open-label arm understand that they were taking a placebo (“What did you think was in the placebo pills?”) and 2) were patients in the no treatment arm disappointed (“Were you disappointed to be in the treatment as usual arm?”) To answer these questions two researchers (TJK, MK) independently extracted the responses to these questions. A third researcher (JPS) compared these extracted responses and a discussion settled two occasions where handwriting that was difficult to interpret. TJK categorized the data using the iterative and emergent methodology of grounded theory. [25], [26] When participants in the placebo arm were asked: “ What did you think was in the placebo pills?” of the 29 who responded, 16 wrote “sugar” (12), “flour” (3) or “calcium” (1),” 6 responded “nothing,” 5 responded “did not know,” 1 responded “symbolic reminder,” and 1 responded “possible test medication.” When participants in the no-treatment arm were asked: “Were you disappointed to be in the treatment as usual arm?” of the 38 who responded, 29 said “no” and only 9 said “yes” or “a little”. We then looked at the responses of the nine who expressed disappointment, to see how they responded to: “What did you like most and least about the trial?” All gave uniformly positive answers such as “I liked that my feeling about the intensity of the problem was validated and was taken seriously…and was able to discuss my IBS,” “the doctor and the nurse were wonderful and accommodating,” “I liked the one-on-one attention with the MD, able to ask questions about IBS with a person trained in the illness; this MD is very kind” (underling in the original). This qualitative data seemed to indicate that, in general, patients understood they were taking placebo and were not overly disappointed in being in the no-treatment arm.

## Discussion

We found that patients given open-label placebo in the context of a supportive patient-practitioner relationship and a persuasive rationale had clinically meaningful symptom improvement that was significantly better than a no-treatment control group with matched patient-provider interaction. To our knowledge, this is the first RCT comparing open-label placebo to a no-treatment control. Previous studies of the effects of open-label placebo treatment either failed to include no-treatment controls [27] or combined it with active drug treatment. [28] Our study suggests that openly described inert interventions when delivered with a plausible rationale can produce placebo responses reflecting symptomatic improvements without deception or concealment.

Our results challenge “the conventional wisdom” that placebo effects require “intentional ignorance.” [29] Our data suggest that harnessing placebo effects without deception is possible in the context of 1) an accurate description of what is known about placebo effects, 2) encouragement to suspend disbelief, 3) instructions that foster a positive but realistic expectancy, and 4) directions to adhere to the medical ritual of pill taking. It is likely our study also benefited from ongoing media attention giving credence to powerful placebo effects.

Both treatment arms were given in a context of a warm patient-provider relationship. It is possible that this relationship had a positive benefit for the patients, and indeed, the no-treatment arm showed improvement. Given that patients in both treatment arms experienced the same frequency and duration of contact time and the content of the interaction was very similar, we believe that the incremental improvement in our open-label arm was due to the addition of open-label placebo treatment. The magnitude of improvement reported by those on open-label placebo treatment was not only statistically significant but also clinically meaningful. The effect size for the primary outcome, calculated as the standardized mean difference (*d*) between the open-label-placebo and no-treatment groups, was 0.79 at endpoint, which is conventionally interpreted as a large effect. [30] At endpoint, we also observed medium sized effects for the differences between placebo and control groups on symptom severity (*d* = 0.53) and quality of life (*d* = 0.40). An improvement from baseline of 50 points on the IBS-SSS reliably indicates meaningful symptomatic improvement. [18] The open-label group improved by 92 points on this measure; in addition, the improvement shown by the open-label placebo group exceeded that shown by the no-treatment group by 46 points. Similarly, an increase of 10 points on the IBS-QoL indicates a clinically meaningful improvement, and we observed an increase of 11 points on this measure for the open-label group. [24] Finally, the percentage of patients reporting adequate relief during the preceding 7 days at the 21-day endpoint (59%) is comparable with the responder rates in clinical trials of drugs currently used in IBS. [31], [32] A recent meta-analysis of double-blind, placebo-controlled trials of alosetron in IBS estimated that 51% of patients treated with alosetron had adequate relief as compared to 38% of patients treated with placebo. [33] Our results were remarkably similar (59% for open-placebo; 35% for no-treatment control), suggesting that open-label placebo in the context of a persuasive rationale may show comparable efficacy to established IBS treatments.

The placebo response in this trial (59% on IBS-AR) was substantially higher than typical reported placebo responses of 30–40% in double-blind IBS pharmaceutical studies. [15] This finding seems counterintuitive. We speculate that it is an indication of the credibility of our open-label rationale. Patients in our study accepted that they were receiving an active treatment, albeit not a pharmacological one, whereas patients in double-blind trials understand that they have only a 50% chance of receiving active treatment. It may be that one hundred percent certainty that one is receiving the “treatment of interest” (in this case open-label placebo) is more placebogenic than a fifty percent probability of receiving an inactive control.

It may be worthwhile to interpreted our study in light of the 2001 landmark meta-analysis of placebo effects and its 2010 expanded and updated version. [34], [35] In the recent analysis, the authors found 202 randomized trials in 60 medical conditions that included placebo and no-treatment groups. When meta-analytically combined, in general, little evidence of clinically meaningful effects of placebo beyond no treatment was found. The meta-analysis, however demonstrated a significantly larger placebo effect for a subset of 28 studies with a specific aim of investigating the placebo effect. Perhaps this subset is most relevant to our study which was also specifically examined placebo effects. Further prospective research will be necessary to clarify under what circumstances and in what conditions one can expect or not expect to find robust placebo responses.

There are intimations in the placebo literature that providers with greater perceived expertise or authority (e.g., physician versus nurse, dentist versus technician) will elicit greater placebo responses. [36], [37] In our study, we found no evidence for significant differences between male physician and female nurse-practitioner.

In addition to its clinical significance, our study has important ethical implications. As mentioned above, evidence indicates that physicians continue to use placebo treatment without transparent disclosure to patients [5], [6] Our results suggest that the placebo response is not necessarily neutralized when placebos are administered openly. Thus our study points to a potential novel strategy that might allow the ethical use of placebos consistent with evidence-based medicine. Minimally, open-label placebo may have potential as a “wait and watch” strategy before prescriptions drugs are prescribed. Further studies of open placebo are merited not only for IBS but for illnesses primarily diagnosed by subjective symptoms and introspective self-appraisal. In sum, our study suggests that for some disorders it may be appropriate for clinicians to recommend that patients try an inexpensive and safe placebo accompanied by careful monitoring before and after prescribing medication. Clearly replication and further research is essential before such a practice could be implemented.

### Limitations

This RCT has several limitations. Most importantly, our sample size was relatively small and the trial duration was too short to obtain estimates of long-term effects. Therefore, the trial could be described as a “proof-of-principle” pilot study. Obviously, replication with a larger sample size and a longer follow-up is needed before clear clinical decisions could be made based on our data.

Other potential limitations of our study may be the issue of report bias (e.g., “wishing to please the experimenter”). However, given the impossibility of double-blind assessment of open placebo versus no-treatment control, the effects of report bias cannot be eliminated. Another related limitation is that patients assigned to no-treatment may have been disappointed, thus inflating the differences between open-label placebo and no-treatment control groups. Importantly, our qualitative check-out data found the no-treatment group experiencing positive support, with 76% of them reporting that they were not disappointed with their assignment. This argues against disappointment being a significant factor. A further possible limitation is that our results are not generalizable because our trial may have selectively attracted IBS patients who were attracted by an advertisement for “a novel mind-body” intervention. Obviously, we cannot rule out this possibility. However, selective attraction to the advertised treatment is a possibility in virtually all clinical trials. In any case, patients in clinical practice are ultimately given choices and it may turn out that open-label placebo will be helpful only for those who elect to try this option. Finally, it could be argued that IBS is a poor illness to study placebo effects because it lacks objective measures. However, there are many serious conditions for which primary outcomes are primarily subjective (e.g. depression, anxiety and chronic pain), and the preponderance of evidence indicates that placebo treatments are most effective for such patient-centered complaints. [1]

In summary, our study suggests that patients are willing to take open-label placebos and that such a treatment may have salubrious effects. Further research is warranted in IBS and perhaps other illnesses to confirm that placebo treatments can be beneficial when provided openly and to determine the best methods for administering such treatments.

## Supporting Information

Checklist S1 (DOC)

Protocol S1 (DOC)
