# Supplementary material for: Placebos without Deception: A Randomized Controlled Trial in Irritable Bowel Syndrome
Source: PLoS One. 2010 Dec 22;5(12):e15591. doi: 10.1371/journal.pone.0015591 (PMC3008733; doi:10.1371/journal.pone.0015591)
Supplement: Protocol S1 — (DOC) [file pone.0015591.s002.doc]

Study Protocol: 9.22.08

**Harnessing Placebo Effects: Non-Deceptive Use of Placebo in IBS**

| Principal Investigator | **Anthony Lembo, MD** | | |
| --- | --- | --- | --- |
| Co-Investigators | **Ted Kaptchuk, Elizabeth Friedlander, PhD, APRN, BC, Norma Sanchez, MA, CCRC, Frank Miller, PhD** | | |
| Mailing Address | **330 Brookline Ave- Dana 501** | | |
| E-Mail Address | [**alembo@bidmc.harvard.edu**](mailto:alembo@bidmc.harvard.edu) | | |
| P.I.’s Telephone | 7-2138 | P.I.’s Pager 38420 | Fax:7-0683 |

**A. Purpose of Protocol**

In this study, we will test whether placebo pills that are truthfully described “as inert substances (placebos), like sugar pills, that have been shown in rigorous clinical tests to somehow produce significant self-healing processes in IBS patients” given in a context of positive expectation and reassurance can produce clinical improvement in patients with IBS. This study is a three-week RCT comparing non-deceptive open label placebo treatment to an observation waitlist control in 80 IBS patients. We believe this study involves no ethical compromise from normative ethical standards and could potentially result in innovative treatment strategy for IBS patients as well as improve our understanding of the placebo effect in clinical trials. This study will ultimately give us preliminary data to support a more rigorous NIH RO1 grant.

**B. Significance and Background for the Study**

1. Significance:

Clinical trials suggest that patients respond to placebo in clinically significant and meaningful ways. Recent basic science research has shown that placebo treatment has a biochemical and neurobiological basis of action similar to that of some pharmacological agents. (Miller & Kaptchuk 2008, Benedetti et al 2005, Pacheco-Lopez et al 2006). But the use of placebo in clinical practice is considered unethical because it involves deception of patients and thus is contrary to informed consent. Currently the effectiveness of placebo randomized controlled trials (RCT) is believed to depend on concealment such that patients believe they may be on a real medication. We are challenging this assumption that placebo is only effective when concealed or involving deception. We hypothesize that open-label non-deceptive administration of an inactive (placebo) pill in the context of patient-practitioner relationship of education, reassurance, positive expectation and confidence can elicit positive clinical outcomes in IBS patients. We propose a RCT to examine whether the non-deceptive administration of placebo pill can improve symptoms in patients with IBS beyond a no treatment waitlist control. The significance and background section below discusses the following issues: 1. placebo responses in RCTs in general and specifically in IBS; 2. mechanistic advances in placebo research including IBS; 3) ethical issues and the use of placebo; 4) a theoretical model of how the components of the placebo response -- including positive expectation, conditioning, adherence effects and a supportive patient-physician relationship --- that do not necessarily require deception and 5) preliminary data suggesting non-deceptive placebo administration can elicit clinical improvement in patients.

2. Placebo Responses in Clinical Medicine:

Clinical research continues to demonstrate that patients in the placebo control arm of RCTs can have dramatic improvements. For example, recent meta-analyses on Parkinson’s disease (Goetz at al 2000), asthma (Joyce et al 2000), social phobia (Oosterbaan et al 2001), migraine (van de Kuy et al 2002), chronic fatigue syndrome (Cho et al 2005), symptom management (e.g., as pain and appetite) in oncology patients (Chvetzoff and Tannock 2003), sleep disorders (McCall et al 2003), restless leg syndrome (Fulda & Wetter 2007) and chronic pain (McQuay et al 1995) have all demonstrated that the response to placebo treatment in RCTs is dramatic and can be clinically significant. In fact, the high response to placebo treatment in RCT is the presumed reason why many widely used medications with well described physiological effects–- including antidepressants; analgesics, anxiolytics, antihypertensives, hypnotics, anti-anginal agents, angiotensin-converting enzyme inhibitors, β-blockers, antihistamines, and nonsteroidal asthma drugs etc. are frequently indistinguishable from placebo in well-designed RCTS. (Temple & Ellenberg 2000, cf. Walach et al 2005)

3. Placebo Responses in IBS:

Similar to other illnesses, placebo responses in clinical trials involving patients with gastrointestinal disorders are high. (Musial et al 2007) Our team has published a meta-analysis (Patel et al 2005) that found that placebo responses in IBS clinical trials ranged between 16.0 to 71.4% with a population-weighted average of 40.2%, 95% CI (35.9, 44.4). More importantly, our team recently demonstrated in a RCT (n=262) that placebo treatment (sham acupuncture) combined with an augmented patient-practitioner context produced ‘adequate relief’ of IBS symptoms in 62% of patients compared to 28% in the wait-list control group (p<0.0001) (Kaptchuk et al 2008). The magnitude of this response is not only statistically significant but also clinically significant and comparable with current FDA approved medications for IBS.

4. Mechanistic Advances in Understanding Placebo Mechanism(s):

Although the physiology of placebo effects is not completely understood, evidence clearly links the release of endogenous opioids with placebo-induced analgesia. (Levine 1978) A recent meta-analysis has shown that when naloxone, an opioid antagonist, is administered by hidden injection, placebo analgesia can be dramatically reduced or even eliminated. (Sauro, 2005) Moreover, using positron emission tomography (PET) to measure endogenous neurotransmission, Petrovic and colleagues (2002) found that both opioid analgesia and placebo analgesia increased cerebral blood flow in brain regions known to be rich in opioid receptors including such as the rostral anterior cingulated cortex (rACC). Likewise, Wager et al (2004), using functional resonance imaging (fMRI), showed that placebo cream was associated with decreased activity in pain-sensitive regions including the insula and ACC when comparing the response to noxious stimuli applied to the control. Building on these and other studies, Zubieta and colleagues (2005, 2006) observed with PET placebo-induced activation of the µ-opioid receptors-mediated neurotransmission endogenous opioid system in subjects with sustained pain.

Importantly, for our hypothesis, recent research has demonstrated that placebo analgesia is not a single entity but rather a spectrum of effects related to the divergence between conscious expectation and unconscious conditioning pathways. (Klinger et al 2006) In an elegant series of experiments, Amanzio and Benedetti (1999) were able to induce different types of placebo analgesia. For example, the placebo response to verbal expectation paired with saline injection could be completely blocked with naloxone. Likewise, morphine conditioning alone (i.e., two separate administrations of morphine without verbal expectation) also produced placebo analgesia that was naloxone-reversible. By contrast, ketorolac (a non-opioid analgesic) conditioning together with expectation produced a placebo response that was only partially blocked by naloxone while ketorolac conditioning alone produced placebo responses that were naloxone-insensitive. Other experiments have also shown that expectations and conditioning effects can be separated and then combined to produced an even greater placebo response than each alone. (Klinger et al 2006, Amanzio and Bededetti 1999) Thus, it seems that placebo responses can be parsed into different mechanisms and can involve conscious expectations and/or unconscious conditioning responses. These studies support our theoretical model that clinical placebo responses are not simply due to expectation or conditioning, but involve multiple pathways.

The recent neuro-chemical and neurophysiologic advances in general placebo effects are echoed in the mechanistic studies of placebo response in IBS patients. For example, using a rectal balloon model of inducing intestinal discomfort in IBS patients, Lieberman et al (2004) showed that placebo analgesia in IBS is related to activation of right ventrolateral prefrontal cortex and is mediated by activation of dorsal anterior cingulated (dACC), an area typical associated with pain unpleasantness. Also important for our proposed study, Vase and colleagues (2005) showed that IBS placebo analgesia does not necessarily involve endogenous opioids but may be related to non-expectation mechanisms. (Vase et al 2005)

5. Ethical Issues on Placebo Use in the Clinical Setting:

The AMA’s House of Delegates recently voted that physicians should consider adopting strategies that “produce a placebo-like effect through the skillful use of reassurance and encouragement.” (American Medical Association 2006). The report also supported the normative ethical position (e.g. Bok 1974) that “in the clinical setting, the use of a placebo without the patient’s knowledge may undermine trust, compromise the patient-physician relationship, and result in medical harm to the patient.” Despite this report, physicians continue to believe in the power of placebo. A recent study found that 96% of 466 surveyed doctors believed that placebo could help patients and up to 40% of the physicians reported that placebos could benefit patients physiologically for certain health problems (Sherman and Hickner 2007). In addition, 48% of respondents to this survey reported giving at least 1 type of treatment in a situation where there was no evidence of clinical efficacy. (Steenhuysen 2008) This study confirmed the findings of others. Hrobjartsson and Norup (2003) in a national survey of 772 Danish physicians found that 86% reported using a placebo treatment at least once within the past year, and 48% reported using placebo treatments >10 times in the past year. In a small Israeli survey (89 respondents) by Nitzan and Lichtenberg (2004), 60% of physicians and nurses reported prescribing placebos. These findings are also consistent with other earlier surveys in Great Britain, Sweden, and New Zealand (e.g. Gray and Flynn 1981, Thomson and Buchanan 1982, Lynoe et al 1993) as well as the early American study by Goodwin and Goodwin (1979). Such surveys suggest that physicians want to utilize placebo effects in clinical practice and sometimes go beyond normative ethical boundaries. These findings suggest that developing a non-deceptive option for placebo usage is an urgent ethical priority.

One potential solution to question of harnessing the placebo response is a direct challenge to the unproven belief that deception is necessary for a placebo to work. While mostly overlooked as an option, there have been such proposals in the past. The psychiatrist Walter Brown (e.g., 1994) has argued that the scientific evidence should make it possible to elicit a placebo effect without deception. In a related argument, the medical philosopher , Brody (1982) maintains that within the context of a supportive, positive, honest and reassuring clinical encounter, can elicit what we usually called placebo effect and can create an “an effective separation of deception and the placebo effect in clinical practice.” While Brody does not suggest open-label placebo is necessary, he proposes that clinical practices can elicit what he calls the “inner pharmacy.” Thus, expectation can be utilized without deceit. Certainly Brody and Brown’s position is not contradicted by more recent scientific evidence. Our experiment is a direct effort to empirically test this hypothesis.

6. Theoretical Rational for Non-Deceptive Placebo Treatment:

Based on our own research and the research of others, we consider the potential for positive clinical improvement from placebo to be a combination of multiple learning processes and environment cues that include: expectation, behavioral conditioning, adherence effects and the effects of a positive, supportive, compassionate and confident patient-physician relationship. We believe these four factors can be combined in a clinical intervention that is behaviorally tied to the taking of an inert substance (placebo pill) to produce what is commonly called “the placebo effect” without deception. We see the contributions of these fours components as follows:

Expectation: Positive expectations have been shown to induce placebo responses across a variety of conditions. (e.g., Benedetti et al 1999, Bausell et al 2006, Mondlock et al 2001, Linde et al 2007) including IBS. A recent study of IBS patients showed that saline infusion of rectal balloon intubation with positive expectation produced analgesia equivalent to rectal lidocaine infusion. (Verne et al 2003, cf. Vase et al 2001) While expectation can arise from just taking a medication in an RCT, multiple experiments have shown that positive expectations alone without any medication can produce significant clinical improvements (e.g., Thomas 1987; Olsson et al 1989). We believe that placebo pills given with positive expectations can have clinical impacts greater than these earlier experiments that just gave positive expectation without inert pills. We believe providing patients with information that demonstrates expectation works will enhance their expectations in a kind of “booth-strap” effect.

Conditioning: As stated in the mechanism discussion of the background section, part of the placebo effect is likely to be due to classical conditioning and is in independent of conscious expectation and belief. For example, almost all patients have experience taking medications that improve their symptoms (e.g., aspirin related compounds for headaches or fever). Thus Pavlovian conditioning may be responsible for some of the placebo response: previous medication (unconditioned response) + pill (conditioned response) allows for a subsequent positive response from the pill alone (conditioned response alone.) In fact, conditioning has been shown to influence immunological, endocrinal, intestinal and other bodily functions. (Pacheco-Lopez et al 2006) Importantly, Amanzio and Benedetti (1999) showed that the placebo effects of conditioning and expectation could be separated and are physiological distinct (expectation is nalaxone reversible and conditioning is not) and at times these effects act synergistically. In a subsequent experiment, this same team was able to show that conditioning is able to override verbal placebo expectations and that conditioning placebo response can happen completely unconsciously to decrease cortisol levels. (Benedetti et al 2003) We believe that a significant proportion of the placebo response in IBS is due to conditioning and that this process can be combined with the positive expectations to elicit placebo effects without deception. We propose that providing patients with information about such conditioning effects will contribute to positive response to our open-label placebo. In addition, explaining that the placebo effect may possibly involve a physiological “triggering” may prevent patients from feeling that the placebo response (or their illness) is “all-in-their head.”

Adherence: Multiple research experiments have shown that patients who adhere to treatment, even when that treatment is a placebo, have better outcomes than those who poorly adherent to placebo. (Horwitz & Horwitz 1993, cf. Granger et al 2005, Irvine et al 1999, Gallagher et al 1993, Czajkowski & Chesney 1990) A meta-analysis of the association between adherence to placebo therapy in eight RCTs with mortality as an endpoint (e.g. as post-myocardial infarction management, primary prevention of cardiovascular disease, and diabetes) showed that those in the placebo arms (19,633 participants) who adhered to taking placebo controls had a better outcome than those who did not take their placebo, (odds ratio 0.56, 95% CI 0.43 to 0.74). (Simpson et al 2006) It is likely that some of this adherence effect is due to a surrogate marker for other health promoting behaviors and attitudes and explanation of it potentially may contribute to positive expectations and conditioning effect needed to elicit the placebo effect.

Patient-Practitioner Relationship: Many studies indicate that the patient-practitioner encounter is a potent factor in health outcomes and a critical potentiating factor for placebo effects. (Kaptchuk 2002, Beck et al 2002, Steward 1995, Ong et al 1995, Kaplan et al 1989, Moerman 2003) For many non-life threatening illness, clear diagnosis, reassurance, and the patient-physician agreement about the nature of the problem hastens recovery or relief of symptoms. (e.g., Findler & Correa 1996, Bass et al 1986). A systematic review of the therapeutic impact of a positive patient-practitioner relationship found 25 eligible RCTs testing this question on physical conditions and found that “physicians who adopt a warm, friendly, and reassuring manner are more effective than those who keep consultations formal and do no offer reassurance.” (Di Blasi et al 2001) Our own recent RCT with IBS patients demonstrated that the patient-practitioner relationship is a significant component of the placebo response. (Kaptchuk et al 2008)

We hypothesize that these four factors – expectation, conditioning, adherence and a supportive patient- practitioner relationship can be operationally bundled together, explained to patients and elicit a clinically significant response to placebo treatment even when the placebo pill is given without deception.

7. Empirical Data Supporting Non-Deceptive Use of Placebos:

Our hypothesis for the efficacy of a non-deceptive placebo treatment is controversial and obviously requires empirical testing. However, the little direct empirical data that exist on this question– and it is very weak data -- supports our hypothesis. The only published study that we are aware of is an observational study of 15 patients with anxiety who were given placebo and told: “many people with your kind of condition have been helped by what are sometimes called ‘sugar pills, and we feel that a so-called sugar pill may help you too. Do know what a sugar pill is? A sugar pill is a pill with no medicine in it at all. I think this pill will help you as it has helped so many others.” (Park and Covi 1965) In this one-week study, only one patient was non-compliant and all 14 completers improved with the average initial patient registering 43% improvement on the anxiety scale used. Doctor rating averaged ‘quite a bit better” with all patients. The embedded qualitative exit interviews with subjects reported statements like: “the pills gave me moral support” or “I think that I had a lot to do with it myself, to be honest. By knowing myself that I had to control myself to keep myself in the right frame of mind.” Obviously, this study is poor evidence, especially given the absence of a treatment as usual control and the small numbers. Nonetheless, it suggests that our hypothesis merits rigorous testing.

A second “placebo dose extension study” provides further empirical support for our hypothesis that clinically-meaningful response to placebo can be produced without concealment or deception. Twenty-six children with attention deficit hyperactivity disorder (ADHS) were randomly assigned to one of two orders of experimental condition: 1) optimal dose of open-label stimulant medication (1 week) then 50% dose (1 week), then 50% dose + placebo (1 week) or 100% medication (1 week), then 50% dose + placebo, then 50% dose. (Sandler and Bodfish 2008) Children and parents/guardians were clearly told the inert nature of the placebo, how placebo pills help children with ADHD and all treatments were open-label to child and parents/guardians and single blind for teachers. In both arms, the symptoms remained the same when placebo was added to the 50% reduction but deteriorated when only 50% medication was received. Somehow full disclosure did not effect subvert a placebo effect. Both of these experiments are small but suggest that it may be possible to elicit a placebo effect without deception.

**C. Purpose of the Protocol**

Dramatic advances have been made in placebo studies. Recent studies suggest that placebo treatment not only can improve how you feel but also alter pathophysiology. Recent research indicates that a placebo response arises from learning processes and environmental cues that include positive expectation, behavioral conditioning, adherence effects, and a positive patient-practitioner relationship. Our own team has just completed a randomized controlled trial (RCT) in 262 patients with irritable bowel syndrome (IBS) which showed that placebo effects are real and have a genuine clinical impact, particularly when combined with a positive patient-practitioner relationship. (Kaptchuk et al 2008) Basic science research has demonstrated that placebo treatment elicits quantifiable changes in neurotransmitters, hormones, immune regulators and regionally specific brain activity that influences peripheral disease processes through plausible physiological mechanisms. (Miller & Kaptchuk 2008, Benedetti et al 2005, Pacheco-Lopez et al 2006) Despite these advances, harnessing such placebo effects clinically remains a challenge in part because of the belief that placebos can only elicit a response if the patient is unaware that they are receiving a placebo, such as in a double blind research protocol. Awareness of a placebo is thought to eliminate its efficacy. The widespread belief that concealment or deception is necessary to harness placebo effect creates a formidable barrier to using inert placebos in clinical practice because it is considered unethical to give a patient in a clinical situation a placebo that is deceptively described as a real drug. This randomized controlled trial seeks to critical challenge this assumption that deception is necessary to directly harness placebo effects by testing whether an open-label non-deceptive administration of placebo pills in the context of a supportive relationship can significantly improve the complaints of irritable bowel syndrome (IBS) patients compared to a treatment as usual control.

Besides the clinical need to seek novel therapies for IBS, this study has an ethical urgency. There are numerous indications that physicians are interested in harnessing placebo effects (American Medical Association 2006) and are already engaged using placebos in an unethical manner with deception. (e.g. Sherman and Hickner 2007, Hrobartsson and Norup 2003, Nitzan and Lictenberg 2004). To date, no study has adequately tested whether deception is required to obtain a placebo response. Our study seeks to test the hypothesis that direct placebo responses can be elicited without deception. In particular we hypothesize that open label placebo treatment without deception in the context of reassurance and positive expectation can provide significant clinical benefits for patients with IBS.

In this study, we will test whether placebo pills that are truthfully described “as inert substances (placebos) that have been shown in rigorous clinical tests to somehow produce significant self-healing processes in IBS patients” given in a context of positive expectation and reassurance can produce clinical improvement in patients with IBS. This study is a three-week RCT comparing non-deceptive open label placebo treatment to an observation waitlist control in 100 IBS patients. We believe this study involves no ethical compromise from normative ethical standards and could potentially result innovative treatment strategy for IBS patients and will ultimately give us preliminary data to support a more rigorous NIH RO1 grant.

**D. Aim:** To determine whether open-label non-deceptive placebo treatment in the context of positive expectation and a supportive and confident patient-practitioner relationship is significantly better than a no treatment + usual care control group matched for time.

**E. Hypothesis**: In IBS patients, a three weeks course of open-label non-deceptive placebo treatment in the context of positive expectation will be superior to no treatment plus usual care control.

#### F. DESCRIPTION OF RESEARCH PROTOCOL

#### Overview: This RCT seeks to determine whether non-deceptive (open-label) administration of placebo pill produces significantly more clinical improvement in IBS patients than an observation control.

#### Overall Design: Eighty patients with IBS will be randomized to a three-week course of either 1: non-deceptive open-label placebo pill in the context of positive expectation + usual care or 2. No treatment + usual care. Both groups will receive an equal amount of time with the practitioner. Patients will be assessed at baseline, mid-point and at the end of three weeks with the standard outcomes measures for IBS.

| Figure 1: Study Flow Chart  Open Label +usual care  (N=40)  No treatment waitlist+ usual care (N=40)  Baseline Evaluation and Randomization  (N=100)  Midpoint  Evaluation  Midpoint  Evaluation  3 Week Endpoint  Evaluation  3 Week Endpoint  Evaluation |
| --- |

Restatement of Research Question: A three-week course of non-deceptive open label placebo treatment will be produce statistically superior outcomes to a no treatment waitlist control group.

Design methodology and intervention:

Description of the non-deceptive placebo treatment arm: The intervention will

be geared to maximizing the contributions of positive expectation,

conditioning, adherence and a supportive and confident patient

practitioner relationship to the placebo response of IBS patients taking an

open-label placebo pill. The first phase of the discussion will be designed

to enhance expectation. Patients will be asked whether they have heard of

the placebo effect and what they think of it. After a brief exchange geared

to supporting any positive feelings the patient may have about placebo effects,

the practitioner will then talk about the evidence supporting the idea that just

taking placebo pills with a positive expectation initiates still poorly understood

effects that can have a profound impact of illness. It will be clearly explained that

placebo are made of "inert" substances like sugar pills but have been show

in clinical trials to improve patient symptoms. The practitioner will discuss

our published meta-analysis that showed on average 42% of patients on placebo treatment improve in IBS RCTs (Patel et al 2005). Furthermore, the patients will be informed about our recent IBS study showing that placebo acupuncture was as effective as current medications for IBS but without the side effects. Also during this discussion, to support expectation and to insure that patients are not conflicted about placebo improvement somehow being a sign that the IBS is “in their heads,” the physician or nurse practitioner will also explain that placebo effects change human physiology and can improve immune, endocrine, and pain mechanisms and that response to placebo is a demonstration of “self-healing capacities.” During the discussion the treating physician or nurse practitioner will naturally and clearly repeat the words that are stated in the informed consent that informs the patient that he/she will receive “an inert substance (the placebo), something like a sugar pill, that has been shown in rigorous clinical tests to somehow produce significant self-healing processes in IBS patients.”

The treatment will also seek to maximize the contributions of adherence

and conditioning to the placebo effect of IBS. Patients will be told that it is critical

to take the pill two times daily with meals and that scientific evidence suggests

that the positive effects of the pill taking will only happen if one is meticulous

about taking the pill. Patients will be asked to describe situations in their life

where taking pills had positive effects in their lives. When such a situation is

remembered, patients will be encouraged to remember this context when

they’re taking their inert medications. To again take any negative

connotation about “the symptoms are in their head” patients will be informed

about how conditioning with pills can “automatic trigger” a conditioning

response like Pavlov’s dog who salivate to a bell. The entire patient-

practitioner encounter will take place in the context of a positive, confident

and supportive relationship. As much as possible, we will follow the protocol

used in our last RCT and to ensure that the interaction includes each of

the following points: the conveyance of a warm and friendly manner,

definite communications of empathy, behaviors that demonstrate

active listening, and an empathic expression of confidence in the likelihood

of improvement when taking pills, even inactive pills. A warm manner will have

the following characteristics: hand shaking, exchange on whether to use first or

last names, eye contact but not over gazing, courtesy, friendliness, orienting

the patient during examination, time for questioning, head nodding, forward

leaning, and uncrossed legs and arms. Empathy will be invoked with the

physician stating how he/she understands the difficulties that people with IBS

live with. Confidence will be fostered as the practitioner explains the

evidence concerning placebo effects. At the end of the first visit the patient will

be given enough placebo for the first half of the study and a mid-point follow-up

will be scheduled. The mid-point follow-up will be similar to the baseline

but abbreviated in time and will also include scheduling a final evaluation.

The entire discussion will take approximately 20 minutes.

Description of the no treatment usual care control arm:

The purpose of this arm is to match the ‘active” arm for time with practitioner. In

the informed consent, patients will be told that they will be randomized into

either the placebo or no treatment waitlist usual care arm. It will be explained,

if they are randomized to the latter, they will continue their regular IBS regimen

and will contribute to the observational IBS group. During the visits, t

he practitioners will seek clear descriptions of the patient’s symptoms

and life-style in relationship to their IBS. This evaluation will be in an identical context of the “active” arm with the exception that no attempt will be made to marshal positive expectation, conditioning response, or confidence of improvement or adherence to their IBS regimen. In order to fill the 20 minutes, the physician or nurse practitioner will provide educational support concerning IBS and go over the patient’s knowledge of IBS and how their life-style, including diet, exercise and stress contributes to their symptoms. We also review their current therapeutic regimen for IBS but we will not attempt to alter it during the study period.

Fidelity of treatment: To monitor fidelity to the time of treatments, the physician or nurse practitioner will put on a timer before the patient enters the room and turn off the timer after she/he leaves and record the time on the chart.

Randomization- Group assignment will be determined based on results from program “Research Randomizer”. Randomization number will be given at time of visit. Allocation to group will be blinded until patient encounters the practitioner.

**D. Data Analysis and Power Analysis:**

Outcome Measures: The following outcome measures will be used in this study. All questions will be asked at baseline, midpoint and endpoint with the exception of IBS-Global Improvement Scale (IBS-GIS) and IBS-Adequate Relief (IBS-AR).

IBS Global Improvement Scale (IBS-GIS): “Compared to the way you felt before you entered the study, have your IBS symptoms over the past 7 days been: 1) “Substantially Worse”, 2)”Moderately Worse, 3)”Slightly Worse”, 4)”No Change”, 5) ”Slightly Improved”, 6)”Moderately Improved” or 7) “Substantially Improved” (Kellow, 2003, Lembo, 2001; Gordon, 2003). A responder was defined as a patient who answered that their symptoms were either “moderately improved” or “substantially improved”. This will be our primary outcome.

IBS Global Symptom Assessment (IBS-GSA): Overall IBS symptoms will be rated by the subject by answering the following global question at the end of each week: Before you started taking the study “medication,” you said that you had [answer (in words) to baseline question 1 will be inserted here]. Compared to how you felt then, how would you rate your overall IBS symptoms during the past week? Possible answers are one of the following: Significantly relieved (+3);

Moderately relieved (+2); A little bit relieved (+1); Unchanged (0); A little bit worse (-1); Moderately worse (-2); Significantly worse (-3).

IBS Adequate Relief: Have you had adequate relief of your IBS symptoms over the past week? Yes or no

IBS Symptom Severity Scale (IBSSSS): The IBS Symptom Severity Scale (IBS-SSS) contains five questions that measure on a 100-point VAS, the severity of abdominal pain, the frequency of abdominal pain, the severity of abdominal distention, dissatisfaction with bowel habits, and interference with quality of life (Francis, 1997). All five components contribute to the score equally yielding a theoretical range of 0-500, with a higher score indicating worse condition. Previous studies have established that scores below 175 represent mild IBS symptoms, 175–300 represents moderate severity, and scores above 300 represent severe IBS (Francis, 1997). The total severity score on the IBS-SSS was treated as the gold standard measure of IBS severity. In the study by Francis et al., a decrease of 50 points correlated with improvement in clinical symptoms and was therefore considered to be a responder.

Abdominal pain/discomfort: Please rate the severity of your abdominal pain or discomfort (0-100)

Feelings of bloating and/or abdominal distention: Please rate the severity of your abdominal bloating or distention over the past week (0-100)

Relief of bloating and/or abdominal distention will be rated by the subject by answering the following question at the end of each week with “Yes” or “No”:

Have you experienced adequate relief of your feelings of bloating and/or abdominal distention during the past week? Additionally, patients will be asked to rate the severity of their abdominal bloating (0-100 scale).

Frequency of bowel movements: Patients will be asked to indicate the average number of bowel movement per day over the past week.

Quality of life questionnaire – specific for IBS (IBS-QoL) The IBS-QoL questionnaire consists of 34 individual questions. The response to each individual question is “not at all”, “slightly”, ”moderately”, “quite a bit”, or “extremely” (alternatively, questions 3, 5-7, 11, 14-15, 17-24, and 30-33 use a response of “a great deal” instead of “extremely”). The response is scored from 1 to 5, where 1 represents “not at all” and 5 represents “extremely / a great deal”. Eight domain scores (i.e. dysphoria, interference with activity, body image, health worry, food avoidance, social reaction, sexual, and relationship) and an overall score are derived from the 34 individual item scores.

**Other Measures:**

Expectations: Subjects will be asked to rate their expectations for improvement at baseline. Subjects will be asked: “At the conclusion of this three week trial how much to you expect your IBS symptoms to improve?” Their responses will be recorded on a 10 point scale with the following anchors: “0=No Improvement” and “10=Complete Relief of IBS Symptoms.”

Patient-Practitioner Relationship rating: The Working Alliance Inventory (WAI) scale will be used to measure the patient’s perception of the relationship with the practitioner. The WAI was developed by Horvath and Greenberg (1989) to measure three components of the patient-practitioner relationship: goal, task and bond. Data shows that WAI is reliably correlated with a variety of counselor and client self-reported measures.

Short Qualitative Description Report: At the completion of the study, after all assessment have been made patients who took the placebo will be asked to briefly describe in their own words responses to the following questions:

1. What do you think about the idea of taking placebo?

2. Did you expect it to work or were you skeptical?

3. What did you think was in the placebo pills?

4. Is there anything you would like to comment on about concerning your experience in the trial?

Subjects on the waitlist will be asked:

1. Were you disappointed to be in the waitlist?

2. What did the like the most and least about the trial?

3. is there anything else you would like to comment on about concerning your experience in the trial?

**Planned Statistical Analyses**

For continuous measures (i.e., IBS-SSS and IBS-QoL), we will conduct analyses of covariance (ANCOVA) controlling for baseline scores to test whether the open-label placebo yields a significantly greater benefit at the three-week endpoint than any improvement occurring in the wait-list control. For categorical measures (i.e., IBS-GIS, IBS-AR), we will conduct chi square tests to determine whether the proportion of responders in the open-label placebo condition significantly exceeds the proportion of responders in the wait-list control. All tests will be two-tailed with alpha set at .05.

**Mediation Analyses**

We also plan to conduct mediation analyses to determine whether any of the hypothesized mechanisms of the open-label placebo response (i.e., expectations, adherence, and the quality of the patient-practitioner relationship as measured by the WAI) can mediate the effects of experimental group assignment. For continuous variables (i.e., IBS-SSS and IBS-QoL), these analyses will take the form of analyses of covariance (ANCOVA), controlling for the influence of the mediators as well as the baseline symptom scores. For categorical outcome variables (i.e., IBS-AR, IBS-GSA, IBS-GIS) we will use logistic regression models. All analyses will be two tailed, with alpha set at .05. If the hypothesized mediators influence outcome, then the association between experimental group assignment (i.e., open-label placebo vs. wait list control) and outcome should significantly diminish or disappear altogether (in the case of complete mediation) once the mediators are added to the model.

**Statistical Power**

Our previous trial of placebo effects in IBS produced effect sizes that ranged from d=.5 to d=.9. Using two-tailed t-tests with alpha set at .05, a total sample size of 80 will have 60% power to detect the lower end of this range and 94% power to detect the upper end of the range. Using the same assumptions but a total sample size of only 50, we would have power between 41% and 88%.

**G. Subject Selection**

| **Subject Selection:**  Patients will be recruited from the BIDMC GI Clinic and through IRB approved advertisements.  **Inclusion/Exclusion Criteria:**  Inclusion Criteria:   1. Only adults (>18 years of age) meeting the Rome III Criteria for IBS will be eligible for entry into the study. 2. Patients with ‘warning symptoms’ (e.g., unexplained weight loss, family history of colon cancer or inflammatory bowel disease, and rectal bleeding) will be required to have had a detailed workup to exclude organic pathology prior to enrolling the patient. 3. Participants will be allowed to continue their IBS medications (e.g., fiber, anti-spasmodics, antidepressants, loperamide, etc.) as long as they had been on stable doses for at least 30 days prior to entering the study and agreed not to change medications or dosages during the trial. 4. Patients will be asked not to make significant changes to their diet during the study. 5. In addition, patients will be required to have active symptoms at the entry of the study as defined by having an IBSSSS score of >150 (0-500 scale).   Exclusion Criteria:   1. Patients will be excluded if they had abdominal surgeries (excluding cholecystectomy, appendectomy, hysterectomy, and hernia repair). 2. History of inflammatory bowel disease (e.g., ulcerative colitis, Crohn’s disease). 3. Recent antibiotic use (within 1 month). 4. Radiation proctitis or other known poorly controlled medical conditions that could interfere with bowel function. 5. Patients will be excluded if they on narcotics or other pain medications (except for non-steroidal anti-inflammatory drugs). 6. Patients will also be required to have active symptoms at the time of entry into the study (score of >150 on the IBS-SSS). 7. Previous experience participating in randomized controlled trials for IBS. |
| --- |

#### H. Possible Benefits

| The patient may or may not benefit from participating in this study. The open placebo may help their Irritable Bowel Syndrome symptoms i.e. their symptoms may be lessened by taking the placebo in the context of positive expectations. The patient’s participation in the study may also help researchers develop more effective medications for treating symptoms of Irritable Bowel Syndrome. |
| --- |

#### I. Possible Risks

| Safety Measurements  The safety of subjects will be monitored at each study visit  - AEs  -Vital signs and body weight  - Physical exam  - Concomitant medication use  *Risks:*  *Subjects may experience normal fluctuations in their IBS symptoms (i.e., abdominal pain or discomfort). But since no active treatment is being given and patients are being evaluated 3 times over 3 weeks by a practitioner experienced in IBS there is minimal risk to the patient.* |
| --- |

## J. Study Location

| Study visits will be conducted in the Division of Gastroenterology, BIDMC in Rabb Rose 1 or alternatively in the GI Motility Center, Stoneman 4 East Campus. |
| --- |

## K. Data Security

| Data will be kept in a locked room that only individuals listed above have access to. Any PHI used by the study sponsor will be assigned a number with the patient’s initials. Files may be kept for a minimum of 6 years, kept in a locked, secured file cabinet or computer in a locked room that only individuals listed above have access to. The sponsor will be contacted prior to destroying files upon archive expiration. |
| --- |
